# Caffeic Acid Release from Inulin Caffeate: A Comparative Study with Spray-Dried Inulin Microparticles Under Gastrointestinal Digestion

**DOI:** 10.3390/antiox15050591

**Published:** 2026-05-07

**Authors:** Patricio Romero-Hasler, Alejandra Quintriqueo-Cid, Begoña Giménez, Eduardo Soto-Bustamante, María Carolina Zúñiga-López, Paz Robert

**Affiliations:** 1 Departamento de Ciencia de los Alimentos y Tecnología Química, Facultad de Ciencias Químicas y Farmacéuticas, Universidad de Chile, Dr. Carlos Lorca Tobar 964, Independencia, Santiago 81380494, Chile; patricio.romero@ciq.uchile.cl (P.R.-H.); aquintriqueo@ug.uchile.cl (A.Q.-C.); 2 Departamento de Ciencia y Tecnología de los Alimentos, Facultad Tecnológica, Universidad de Santiago de Chile, Av. Víctor Jara 3769, Estación Central, Santiago 9170124, Chile; bego.gimenez@usach.cl; 3 Departamento de Química Orgánica y Fisicoquímica, Facultad de Ciencias Químicas y Farmacéuticas, Universidad de Chile, Dr. Carlos Lorca Tobar 964, Independencia, Santiago 81380494, Chile; esoto@ciq.uchile.cl; 4 Departamento de Química Inorgánica y Analítica, Facultad de Ciencias Químicas y Farmacéuticas, Universidad de Chile, Sergio Livingstone, 1007, Independencia, Santiago 8380492, Chile; mczuniga@ciq.uchile.cl

**Keywords:** inulin, chemical functionalization, caffeic acid, in vitro simulated digestion, microencapsulation

## Abstract

This study compared the physicochemical properties of inulin caffeate, where caffeic acid (CA) was covalently bound to inulin (CA-Inu, degree of substitution: 0.07), with CA spray-dried inulin microparticles (Mp(CA/Inu)), as well as release profiles of CA from CA-Inu and Mp(CA/Inu) under in vitro simulated gastrointestinal digestion. Encapsulation by spray drying was optimized using a Central Composite Design, achieving an encapsulation efficiency of 98%. CA was rapidly released from Mp(CA/Inu) during the oral and gastric phases, followed by a slight decrease in the intestinal phase due to interaction with digestive enzymes. In contrast, no release of CA was detected from CA-Inu during the oral and gastric phases. Approximately 30% was released in the intestinal phase, with a further increase in the colonic phase, especially in the presence of inulinase. This strategy, based on the covalent binding of phenolic compounds to biopolymers, can promote targeted delivery in the later stages of gastrointestinal digestion.

## 1. Introduction

Currently, there is a significant trend in the food industry focused on innovating polyphenol-rich ingredients for the formulation of healthier food products. Nevertheless, to capitalize on their advantageous effects, it is essential that they preserve their biological activity despite exposure to environmental, food-related, and digestive conditions. Caffeic acid (3,4-dihydroxycinnamic acid; CA), a polyphenol found in coffee, potatoes, berries, honey, olives, carrots, and other sources [1], has shown local therapeutic effects regarding inflammatory bowel disease (IBD) by reducing several pro-inflammatory factors. During the colonic phase, it exhibits the capacity to modulate the microbiota, thereby promoting the production of short-chain fatty acids such as butyric acid, known for its anti-inflammatory properties [2,3] and reducing endotoxemia [4]. However, CA is particularly prone to oxidation at neutral and alkaline pH [5], which limits its ability to exert beneficial effects. Furthermore, it has been demonstrated that CA may interact with digestive enzymes [6,7] and binds to bile salts, contributing to its reduced presence during simulated intestinal digestion [8].

In this context, encapsulation technology by spray-drying is a common strategy for protecting bioactive compounds, such as CA, and enabling their targeted delivery. Spray-drying exhibits a wide range of advantages that make it highly applicable in the food industry, such as its simplicity, low cost, equipment availability, ease of scaling up, and high reproducibility [9]. However, spray drying is more of an immobilization method than an encapsulation one, which makes it necessary to optimize the process in order to achieve high encapsulation efficiency. In addition, high inlet air temperatures are typically used, which can cause the degradation of temperature-sensitive bioactive compounds [9]. On the other hand, its performance is highly dependent on the selection of an appropriate biopolymer, which can limit the achievement of the desired outcomes. Inulin (Inu) has been successfully used as a biopolymer for spray-drying encapsulation of various polyphenolic compounds, including quercetin [10,11,12], epicatechin [10], naringenin [11], olive leaves extract [13], phenol-rich blackcurrant juice [14], phenol-rich Red Dragon Fruit Kombucha [15], anthocyanin-rich strawberry by-products [16], and phenol-rich spent coffee ground extracts [17]. Inulin is a linear chain of β-(2-1) glycosidic linked D-fructose units, typically capped by an α-(2-1) D-glucopyranose unit (G-Fn) [18], exhibiting a wide range of molecular weights depending on the vegetal source, ranging from 50 to 13,000 Da (polymerization degree from 10 to 60) [19]. Human digestive enzymes are unable to digest Inu due to its β-(2,1) glycosidic linkages [20]. Its wide range of molecular weights, indigestibility, and prebiotic effect [21,22,23] make it attractive for several food and pharmaceutical applications, including site-specific vehicles for transporting drugs/bioactive compounds to the colon [24,25].

Another less explored strategy for the targeted delivery of phenolic compounds during digestion involves their immobilization in biopolymers, such as Inu, through chemical functionalization. Esterification between the carboxylic group of CA and the hydroxyl groups of Inu is expected to form a hydrolyzable bond susceptible to both pH and enzymatic action in the gastrointestinal tract, enabling the release of the polyphenol. Consequently, this study hypothesizes that the release of CA in the upper gastrointestinal tract (during the oral and gastric phases) will be reduced by functionalizing Inu with CA, with primary delivery occurring upon reaching the intestine and colon. Inulin has been functionalized with several molecules using different chemical approaches to yield neutral, anionic, and cationic compounds [26,27]. In this context, Inu has also been functionalized with various phenolic compounds, such as *p*-hydroxycinnamic acid, vanillic acid, and ferulic acid [28,29,30]. However, the performance of these conjugated compounds as oral delivery systems for phenolic compounds has not yet been studied.

The aim of this study was to evaluate the release profile of CA from CA-functionalized Inu (inulin–caffeate, CA-Inu) during in vitro simulated gastrointestinal digestion, and to compare it with the release profile from spray-dried CA Inu microparticles (Mp-(CA/Inu)). Furthermore, the functionalization of Inu with CA was confirmed by proton nuclear magnetic resonance (^1^H-NMR), and the degree of substitution was determined. Both CA-Inu and Mp-(CA/Inu) were also characterized in terms of their physicochemical, thermal, and morphological properties.

## 2. Materials and Methods

### 2.1. Materials

Caffeic acid (3-(3,4-dihydroxyphenyl)-2-propenoic acid, CA) 98%, triphenyl phosphine (TPP) 98%, diisopropyl azodicarboxylate (DIAD) 98% and Amberlyst 15 were obtained from AK Scientific (Union City, CA, USA). Inulin Orafti^®^ HP (degree of polymerization, DP ≥ 23, Inu) was obtained from Blumos S.A. (Santiago, Chile). Pepsin was obtained from porcine gastric mucosa (P6887, 1162 ± 247 U/mg), pancreatin from porcine pancreas (P7545, 8 × USP specifications, 5.8 ± 0.7 U/mg trypsin and 60 ± 3 U/mg lipase), and bile extract (B8631), triphenylphosphine, sodium methoxide, guanidine nitrate, 2,2′-Azobis(2-methylpropionamidine) dihydrochloride (AAPH), and 6-Hydroxy-2,5,7,8-tetramethylchroman-2-carboxylic acid (Trolox) were purchased from Sigma-Aldrich (Santiago, Chile). The fructanase mixture (E-FRMXPD, 420 U/mg) was obtained from Megazyme Neogen (Santiago, Chile). Sodium carbonate, sodium hydroxide, sodium sulfate, acetic anhydride for analysis, dichloromethane for analysis, ethyl acetate for analysis, N-N-dimethylformamide, formic acid for analysis, fluorescein sodium and Folin reagent were acquired from Merck Millipore (Santiago, Chile). Dimethylsulfoxide-d6 (d_6_-DMSO, 99.8%) was obtained from Zeotope, Zeochem AG (Rüti, Switzerland). Finally, methanol for HPLC gradient and ethanol absolute for analysis were purchased from AppliChem Panreac (Santiago, Chile).

### 2.2. Synthesis of Inulin Caffeate

**Figure 1:**
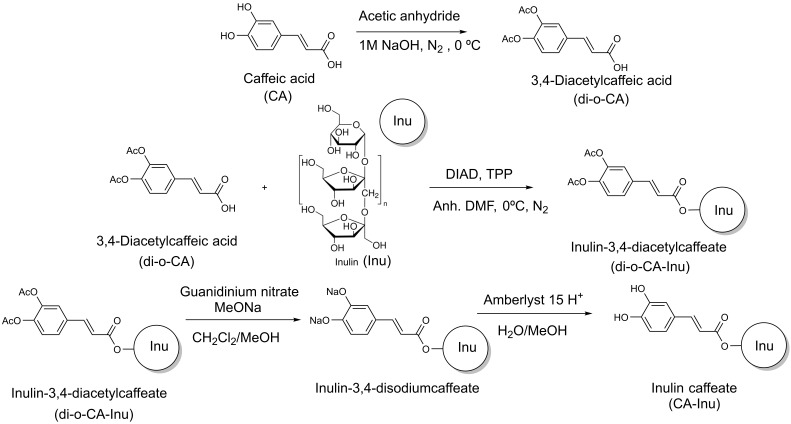
Synthetic pathway used for the synthesis of inulin caffeate (CA-Inu).

The synthesis of caffeic acid-functionalized inulin (inulin caffeate, CA-Inu) was performed in three steps. Initially, CA was protected by acetylation with acetic anhydride (di-O-CA) [31]. Then, the protected CA was esterified to Inu using a modified Mitsunobu coupling (di-O-CA-Inu) [32]. Finally, CA-Inu was obtained by deprotecting the diacetylcaffeic moieties with guanidinium/guanidine system [33] (Figure 1).

#### 2.2.1. Synthesis of 3,4-Diacetylcaffeic Acid

In a 250 mL double neck round-bottom flask with a magnetic stirrer, 10 g of CA (55.5 mmol, 1 equiv.) were weighed. The flask was sealed with a rubber septa and purged with nitrogen. Then, 150 mL of 1 M NaOH were added with a syringe. The mixture was stirred in an ice bath for 5 min, and 20 mL of acetic anhydride were added, which yielded a white precipitate. After 30 min, the mixture was filtered through a glass frit and washed with cold water. The product (3,4-diacetylcaffeic acid, di-O-CA) was recrystallized in 150 mL of hot ethanol, filtered, and rinsed with cold ethanol.

^1^H-NMR, δ ppm, d_6_-DMSO: H_1_: 12.78 (s, 1H, –COOH); H_2_: 7.67 (d, *J* = 2.2 Hz, 1H, Ar-H); H_3_: 7.64 (dd, *J* = 2.2 Hz, *J* = 7.4 Hz, 1H, Ar-H); H_4_: 7.58 (d, *J* = 16.1 Hz, 1H, =CH–); H_5_: 7.32 (d, *J* = 8.3 Hz, 1H, Ar-H); H_6_: 6.54 (d, *J* = 16.0 Hz, 1H, –CH=); H_7_: 2.30 (s, 3H, –OOC–CH_3_); H_8_: 2.31 (s, 3H, –OOC–CH_3_).

#### 2.2.2. Synthesis of Inulin-3,4-Diacetylcaffeate

Dry Inu (5 g, 30.8 mmol, 2 equiv. based on the fructosyl unit), 3,4-diacetylcaffeic acid (4.07 g, 15.4 mmol, 1 equiv.), and triphenylphosphine (TPP, 4.44 g, 16.94 mmol, 1.1 equiv.) were each dissolved in anhydrous N,N-Dimethylformamide (DMF) (60 mL, 40 mL, and 40 mL, respectively) at 80 °C, and then they were transferred into a flame-dried 250 mL double neck round-bottom flask sealed with rubber septa, purged with nitrogen and stirred in a salt-ice bath for 10 min. DIAD (3.33 mL, 3.43 g, 16.94 mmol, 1.1 equiv.) were added dropwise with a syringe. The mixture was left to reach room temperature and was stirred under nitrogen overnight. Water (50 mL) was added to quench the unreacted TPP/DIAD. The DMF was removed from the reaction mixture by rotary evaporation at 60 °C and 20 mbar (Rotavapor^®^ R-100, Büchi, Switzerland). The concentrated mixture was dissolved in water, and the organic by-products were extracted with ethyl acetate. The aqueous fraction was rotary evaporated. The remaining solid was dissolved in hot water and then poured dropwise over an excess of vigorously stirred cold acetone to reprecipitate inulin-3,4-diacetylcaffeate (di-O-CA-Inu).

^1^H-NMR, δ ppm, d_6_-DMSO: H_1_: 7.67 (m, 3H, Ar-H, =CH–); H_2_: 7.33 (d, *J* = 8.4 Hz, 1H, Ar-H); H_3_: 6.69 (d, *J* = 16.3 Hz, 1H, =CH–); H_4_: 5.15 (d, *J* = 5.4 Hz, 1H, –OH); H_5_: 4.7 (d, *J* = 6.6 Hz, 1H, –OH); H_6_: 4.61 (s, 1H, –OH); H_7_: 4.07 (m, –CH–); H_8_: 3.81 (m, –CH–); H_8_: 3.52 (m, –CH–); H_9_: 2.29 (s, 3H, –OOC–CH_3_); H_10_: 2.29 (s, 3H, –OOC–CH_3_).

#### 2.2.3. Synthesis of Inulin Caffeate

Inulin-3,4-diacetylcaffeate (di-O-CA-Inu, 5 g) was dissolved in 100 mL methanol, and added dropwise to a strongly stirred guanidine/guanidinium solution (2.488 g of guanidine nitrate, 4 mL of 1M MeONa in MeOH, and 200 mL of CH_2_Cl_2_/MeOH 1:9) [34]. The phenolate form of inulin-(E)-3-(3,4-dihydroxyphenyl) acrylate precipitated as a yellow solid, which, after 15–30 min, was centrifuged at 4460× *g* for 20 min, rinsed with methanol and vacuum-dried. The solid was dissolved in water:methanol (1:1 *v*/*v*), and Amberlyst 15 in its acid form was slowly added until a pH of 6–7 was reached. The Amberlyst 15 was then filtered off and rinsed. The solution was rotary evaporated to dryness using a rotary evaporator. The resulting solid was then dissolved in deionized water (300 mg per 20 mL tube) and dialyzed against 2 L deionized water using Pur-A-Lyzer™ Mega dialysis tubes (Sigma-Aldrich, Rehovot, Israel) with a molecular weight cutoff (MWCO) of 1 kDa for 2 days, changing the external reservoir water twice. Finally, the dialyzed sample (~20 mL) was frozen at −20°C overnight and lyophilized at 0.2 mbar for 2 days in a freeze dryer (Lyovapor L-200, Büchi, Switzerland) to obtain CA-Inu.

^1^H-NMR, δ ppm, d_6_-DMSO: H_1_: 9.58 (s, 1H, Ar-OH); H_2_: 9.13 (s, 1H, Ar-OH); H_3_: 7.50 (d, *J* = 15.6 Hz, 1H, =CH–); H_4_: 7.05 (s, 1H, Ar-H); H_5_: 7.01 (s, 1H, Ar-H); H_6_: 6.76 (d, *J* = 8.0 Hz, 1H, Ar-H); H_7_: 6.32 (d, *J* = 15.7 Hz, 1H, =CH–); H_8_: 5.15 (d, *J* = 5.4 Hz, 1H, –OH); H_9_: 4.71 (d, *J* = 6.6 Hz, 1H, –OH); H_10_: 4.62 (s, 1H, –OH); H_11_: 4.05 (m, –CH–); H_12_: 3.81 (m, –CH–); H_13_: 3.52 (m, –CH–).

### 2.3. Characterization of Inulin Caffeate

CA-Inu was characterized according to the following parameters.

#### 2.3.1. Proton Nuclear Magnetic Resonance

^1^H-NMR spectra were obtained from a Bruker AVANCE DRX 300 (Karlsruhe, Germany) spectrometer operating at 300.13 MHz. Measurements were carried out at a probe temperature of 300 K, using d_6_-DMSO. The sample was treated overnight with molecular sieves to eliminate the presence of water. The sieves were removed before acquiring the spectra.

#### 2.3.2. Attenuated Total Reflection Fourier Transform Infrared Spectroscopy (ATR-FTIR)

Infrared absorption spectra were measured on the neat solid samples using a single reflection germanium prism ATR (Attenuated Total Reflectance) accessory (Miracle, PIKE, Madison, WI, USA) in a Fourier transform infrared spectrometer (Nicolet iS50R, Thermo Fisher Scientific, Madison, WI, USA) covering the spectral range from 4000 to 800 cm^−1^. The spectra were corrected by intrinsic ATR effects on band absorption intensity and peak shift effects using the spectrometer software (OMNIC v9.7.39).

#### 2.3.3. Degree of Substitution (DS)

DS was determined by ^1^H-NMR spectroscopy and Folin–Ciocalteu colorimetric assay.

^1^H-NMR: Two zones were used between 3.0 and 4.4 ppm, corresponding to the alkyl protons of the fructosyl moieties (7 protons total), and the range 6.0 to 8.0 ppm, corresponding to three aromatic protons and two allylic protons of the CA moiety (5 protons total). DS can range from zero (no substitution) to three (esterification of the three hydroxyls of the fructosyl moiety) (Equation (1)):

(1) $${DS}_{NMR}=\frac{Area\;allylic+aromatic\;protons}{5}\times \frac{7}{Area\;allylic\;protons}$$

Folin–Ciocalteu: The total phenolic content of CA-Inu was determined through a modified method at a milder pH [35]. Briefly, 150 μL of the sample were added to 750 μL of Folin reagent (2 N) diluted in Milli-Q water at a 1:30 ratio (67 mM) and mixed for 5 min. Then, 600 μL of Na_2_CO_3_ 0.75% *w*/*v* was added, and the mixture was incubated for 2 h in the dark at room temperature. Transmittance was measured at a wavelength of 760 nm in a dual-beam UV–Vis spectrophotometer (Halo DB-20, Dynamica, UK). The DS was obtained by quantifying the amount of caffeoyl groups using a caffeic acid calibration curve (2.5 to 100 ppm, R^2^ = 0.999).

### 2.4. Encapsulation of Caffeic Acid with Inulin by Spray Drying

A Central Composite Design (CCD) with 4 central points (α = 1.21), 4 experimental points and 4 axial points was applied to optimize the CA encapsulation with Inu by spray-drying. The independent variables were the CA content (0.27 to 0.93 g per 100 g infeed) and the inlet air temperature (127 to 193 °C), which falls within the range of temperatures generally reported for spray drying (110–220 °C) [36]. The dependent variable was the encapsulation efficiency (EE) of CA.

The infeed dispersion (100 g) was prepared by dissolving Inu (10 g) in 80 mL of deionized water under magnetic stirring and heated up to 85 °C in a water bath until total solubilization. CA (0.27 to 0.93 g) was dissolved in hot ethanol (9.73 to 9.07 g), and then it was added to the hot Inu solution and stirred for 5 min at 85 °C. The infeed was fed into a spray dryer (Mini Spray dryer B-290, Büchi, Switzerland) at 60 °C, stirring at 250 rpm (Heidolph MR-Hei, Heidolph Inst. GmbH, Schawabach,Germany). The spray-drying process conditions were: inlet airflow of 600 L/h, atomization pressure of 2 psi, feeding rate of 1 mL/min, and the inlet air temperature was set according to experimental design.

Response surface methodology was applied to optimize (maximize) the EE of CA. Analysis of variance (ANOVA), lack of fit test, and determination of the regression coefficients were performed using the software Statgraphics (7.0 version, Manugistics Inc., Rockville, WI, USA). Data were fit to a second-order regression model (Equation (2)), considering linear, quadratic, and cross-product forms of the inlet air temperature and CA content:

(2) $$EE=\beta_{0}+\beta_{1}X_{1}+\beta_{2}X_{2}+\beta_{12}X_{1}X_{2}+\beta_{11}X_{12}+\beta_{22}X_{22}+\epsilon$$

where *EE* was the estimated response; $X_{1}$ and $X_{2}$ are the levels of the independent variables; $\beta_{0}$ was the intercept term; $\beta_{1}$ and $\beta_{2}\;$ the linear coefficients; $\beta_{12}$ the interaction coefficient; $\beta_{11}$ and $\beta_{22}$ the quadratic coefficients and ϵ the error.

#### Encapsulation Efficiency

Total caffeic acid: Caffeic acid–inulin microparticles (Mp(CA/Inu); 100 mg) were dispersed in 2 mL of methanol, vortexed (FineVortex, FinePCR, Gunpo-si, Republic of Korea), sonicated (Elmasonic E 30 H, Elma-Hans Schmidbauer GmbH, Singen, Germany) for 5 min, and centrifuged (Hettich Universal 320R, Tuttlingen, Germany) at 9060× *g* for 10 min. The pellet was extracted twice following the same methodology. The supernatants were combined and filled to 10 mL before being injected into the HPLC.

Surface caffeic acid: Mp(CA/Inu) (100 mg) was dispersed in 4 mL of ethyl acetate:ethanol (2:1 *v*/*v*), gently stirred for 1 min, and filtered in a 0.22 μm PTFE syringe filter (Macherey-Nagel GmbH, Düren, Germany). The filtrate (2 mL) was vacuum-dried, redissolved in methanol, and injected into the HPLC.

Caffeic acid chromatographic analysis: CA quantification was performed by HPLC using an Alliance e2695 chromatograph (Waters, Milford, MA, USA), equipped with a photodiode-array detector (Waters 2998, Waters, Milford, MA, USA) and a C18 column (5 μm, 4.6 mm i.d. ×250 mm, Symmetry, Waters, Dublin, Ireland). The mobile phases consisted of solvent A (Milli-Q water) and solvent B (methanol with 0.4% formic acid), with a flow rate of 0.8 mL/min. The gradient elution profile was set as follows: 30% B (0 to 3 min), 50% B (3 to 5 min) and 30% B (5 to 10 min). CA was quantified using a calibration curve (1 to 100 µg/mL, R^2^ = 0.999). Chromatograms were recorded at 325 nm with an injection volume of 20 µL.

The EE of CA was determined using Equation (3):

(3) $$EE\;\left(\%\right)=\;\frac{Total\;caffeic\;acid\;-Surface\;caffeic\;acid}{Total\;caffeic\;acid}\;\times 100$$

### 2.5. Characterization of Inulin Caffeate and Caffeic Acid–Inulin Microparticles

CA-Inu and Mp(CA/Inu) were characterized according to the following parameters.

#### 2.5.1. Wide-Angle X-Ray Scattering (WAXS)

WAXS patterns for CA-Inu and Mp(CA/Inu) were obtained in a laboratory beamline SAXS/WAXS/GISAXS system (SAXSPoint 2.0, Anton Paar, Graz, Austria), using a Cu-Kα microfocus source (Anton Paar, Primux 100) with a copper anode and Astix optics, obtaining a point-collimated parallel beam with wavelength CuKα = 1.5418 Å. It has a 2D detector (Dectris, Eiger R 1 M) with a sample-to-detector distance ranging from 115 mm (WAXS) to 562 mm (SAXS). The samples (CA-Inu and Mp(CA/Inu)) were confined in sealed 1.0 mm diameter special glass capillaries (Charles Supper Company, Natick, MA, USA). Data acquisition was performed using SAXSDrive software v2.02.295.15850 (Anton Paar, Austria), capturing a single frame with a 900 s exposition at a sample-to-detector distance of 115 mm. The data was subsequently reduced using SAXSAnalysis software v4.20.048.15546 (Anton Paar, Austria) following transmission correction and background subtraction, resulting in intensity vs. 2θ X-ray scattering patterns.

#### 2.5.2. Thermal Analysis

The thermal behavior of CA-Inu and Mp(CA/Inu) was studied with a TA Q20 differential scanning calorimeter (TA Instruments-Waters LL, Delaware, DE, USA). The samples were confined in Tzero^®^ aluminum pans (TA Instruments-Waters LL, Delaware, DE, USA) and, both the sealed and non-sealed pan, were measured in Modulated Differential Scanning Calorimetry (MDSC) mode with a 1.5 °C/min heating rate using a sinusoidal temperature modulation of 1.5 °C amplitude and 90 s period. The thermograms were processed with Universal Analysis v4.5A software (TA Instruments-Waters LL, Delaware, DE, USA).

#### 2.5.3. Morphology

The morphology of CA-Inu and Mp(CA/Inu) was evaluated by scanning electron microscopy (SEM) using a high-resolution scanning electron microscopy (FE-255 SEM, Inspect-F50, FEI, Eindhoven, The Netherlands) with a secondary electron detector operated at 2 kV. The samples were gold sputtered with a Sputter Coater (Cressington model 108, Ted Pella Inc., Redding, CA, USA) equipped with a thickness controller (Cressington MTM-20) at a thickness of 10 nm. Images were captured and processed with xT Microscope Control software (FEI, Hillsboro, OR, USA), version 4.1.13.2167.

#### 2.5.4. Moisture, Hygroscopicity, and Water Activity

The moisture content was determined gravimetrically [12]. Samples (CA-Inu and Mp(CA/Inu)) were weighed in a clock glass and dried in a forced convection oven (BE 500, Memmert^®^, Schwabach, Germany) at 105 °C until a constant mass was reached. The water activity of the samples (1 g) was measured by determining the dew point (Hygrolab2, Rotronic, Hauppauge, NY, USA) at 20 ± 3 °C. The hygroscopicity was measured gravimetrically [12]. Samples (100 mg) in a clock glass were placed in a desiccator with Na_2_SO_4_ (81% relative humidity) for one week at 25 °C.

#### 2.5.5. Solubility

The solubility of CA-Inu and Mp(CA/Inu) was determined gravimetrically [12]. Samples (100 mg) were dispersed in distilled water (10 mL), stirred for 5 min, and centrifuged at 1100× *g* for 5 min. An aliquot (2.5 mL) was dried at 105 °C in an oven (BE 500, Memmert^®^, Schwabach, Germany) for 5 h. The weight of the dried soluble solid was recorded, and solubility was expressed as a percentage.

#### 2.5.6. Particle Size

The particle size and size distribution of Mp(CA/Inu) were analyzed by light scattering using a laser scattering particle size analyzer (Partica LA-960, Horiba, Kyoto, Japan; 650 nm laser diode). The microparticles were measured as dry powder using PowderJet (Horiba, Kyoto, Japan) with compressed air at 0.30 MPa (3 bar). The particle size was expressed as the mean particle size (D_4,3_).

#### 2.5.7. Antioxidant Activity

The oxygen radical absorbance capacity (ORAC) was measured for CA-Inu and Mp(CA/Inu) [37]. Fluorescein sodium (65.5 nM) and 2,2′-Azobis(2-methylpropionamidine) dihydrochloride (AAPH, 150 mM) were dissolved in phosphate buffer (pH 7.4). A standard curve of Trolox was prepared in the range of 15 to 150 µM (normalized area = 15.7·[Trolox] (µM) + 393.6, R^2^ = 0.9986). The assay was performed in a 96-well dark microplate in a multi-mode reader (Synergy HTX, BioTek, Winooski, VT, USA) with fluorescence filters (excitation and emission wavelengths of 485 and 520 nm, respectively). Trolox curve points, 25 µL of each sample, and blanks were added to the microplate in quadruplicate. Then, 150 µL of fluorescein were added to the samples, shaken for 50 s, and incubated for 3 min at 40 °C. Subsequently, 25 µL of AAPH were added, and fluorescence decay was recorded every minute at 40 °C. The area under the fluorescence curve for each sample was integrated and interpolated into the Trolox calibration curve. The antioxidant capacity was expressed as µmol Trolox/mg CA.

In the presence of antioxidants (*AOX*), the decrease in fluorescence shows an induction time (*t_i_*), given by Equation (4) [38,39]:

(4) $$t_{i}=\frac{n\times \left[AOX\right]}{R_{i}}$$

where the parameter *n* is the stoichiometric number, which is defined as the moles of radicals scavenged by each antioxidant molecule, [*AOX*] is the antioxidant concentration, and *R_i_* is the rate of radical generation from the APPH initiator.

### 2.6. Simulated In Vitro Digestion

CA-Inu and Mp(CA/Inu) underwent successive oral, gastric, and intestinal digestion phases according to the INFOGEST protocol 2.0 [40]. CA was also subjected to digestion for comparative purposes. Gastrointestinal digestion was carried out in a 50 mL water-jacketed beaker maintained at 37 °C with a JSRC-13C recirculating chiller (JS Research Inc., Gongju, Republic of Korea), and stirred with a Heidolph MR-Hei Standard magnetic stirrer (Heidolph Instruments GmbH, Schwabach, Germany).

Oral phase: CA (3.68 mg), CA-Inu (53 mg, containing 3.68 mg CA) and Mp(CA/Inu) (100 mg, containing 3.68 mg of CA) were dispersed in water (4.9 mL), and 4 mL of simulated salivary fluid (SSF) were added. Then, 25 µL of 0.3 M CaCl_2_ and 0.975 mL of water were added to achieve a final volume of 10 mL. The mixture was incubated at 37 °C for 2 min at 250 rpm. At the end of the oral digestion, a 500 µL aliquot was extracted.

Gastric phase: To the remaining 9.5 mL of the oral phase, 7.6 mL of simulated gastric fluid (SGF) was added, and the pH was adjusted to 2.0 with 1 M HCl. Then, 4.8 µL of 0.3 M CaCl_2_ and 11.62 mg of porcine pepsin (2000 U/mL gastric phase) were added. Distilled water was added to complete the total volume of 19 mL for gastric digestion. The mixture was then incubated at 37 °C for 2 h at 250 rpm. Aliquots (500 µL) were taken every 30 min.

Intestinal phase: To the remaining 18 mL of gastric digest, 7.65 mL of simulated intestinal fluid (SIF) and 36 µL 0.3 M CaCl_2_ were added, adjusting the pH to 7 using 1 M NaOH. Then, 482 mg of pancreatin (trypsin activity of 100 U/mL intestinal phase), and 420 mg of bile were dispersed separately in SIF and added to the intestinal digestion. Distilled water was added to bring the final volume to 36 mL. The mixture was incubated at 37 °C for 2 h at 250 rpm. Aliquots (500 µL) were taken at various time points.

Colonic phase: The colonic phase is not included in the INFOGEST protocol. It was simulated by incubating the final intestinal digest with inulinase (Fructanase^®^, Megazyme Neogen, Santiago, Chile) instead of fecal inoculum, as follows. After the intestinal phase, 10 mL of the digested sample were transferred to a 25 mL capped laboratory bottle. Then, 125 U of inulinase (5 U/mg Inu) was added to the mixture, which was subsequently incubated for 24 h at 37 °C using a JSSI-100C orbital shaker (JS Research, Korea). Aliquots of 250 µL were taken at 1, 3, 6, 18, and 24 h.

#### Quantification of Caffeic Acid Released During Digestion

The quantification of released CA in the digestion aliquots involved gentle centrifugation at 2430× *g* to avoid cracking the remaining microparticles. An aliquot of the supernatant (100 µL) was diluted with 900 µL of methanol, acidified with 2.5 µL of formic acid, and vortexed. The samples were then centrifuged at 14,000× *g* to remove any remaining Inu or bile residues, filtered through a 13 mm 0.22 μm pore size PTFE syringe filter (Macherey-Nagel GmbH, Düren, Germany), and injected as described in the section Encapsulation Efficiency.

## 3. Results

### 3.1. Synthesis and Characterization of the Caffeic Acid-Functionalized Inulin (Inulin Caffeate)

Steglich and Mitsunobu esterification have been reported to prepare caffeates with different monomeric substrates [41]. However, this approach was unsuccessful to obtain CA-Inu directly from CA and Inu. As an alternative, CA has been protected by acetylation in the synthesis of other caffeates [31,42], such as inulin cinnamate, *p*-hydroxicinnamate, vanillate and inulin ferulate [28,29,30]. This strategy was selected due to the rapid and easy removal of acetyl moieties without affecting the CA-Inu. Steglich coupling with the protected CA yielded several side reaction products compared to Mitsunobu. Therefore, the functionalization of Inu with CA was performed in three steps: protection of CA to obtain 3,4-diacetylcaffeic acid (di-O-CA), followed by Mitsunobu esterification of Inu to inulin-3,4-diacetylcaffeate (di-O-CA-Inu), and finally deprotection of the hydroxyl groups to obtain CA-Inu (Figure 1).

#### 3.1.1. Proton Nuclear Magnetic Resonance

**Figure 2:**
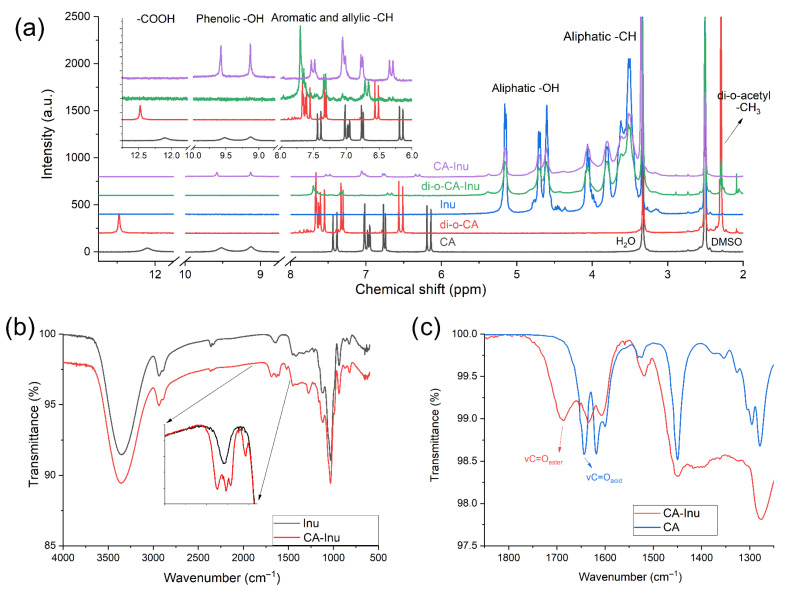
^1^H-NMR (300 MHz in d_6_-DMSO at 20 °C) and ATR-FTIR (single-reflection Ge prism, 25 °C) spectra. (**a**) ^1^H-NMR spectra for inulin (Inu), caffeic acid (CA), inulin di-o-acetylcaffeate (di-O-CA-Inu) and inulin caffeate (CA-Inu). (**b**) Comparison of Inu and CA-Inu showing the presence of new signals arising from the caffeate moiety. (**c**) Comparison of CA-Inu and CA, showing the carbonyl stretching signal shift after esterification.

The ^1^H-NMR spectra are summarized in Figure 2a and Figure S1. CA and the protected CA with the acetyl groups are in good agreement with the literature [31]. The acetylation of caffeic acid (di-O-CA) caused a shift in several signals in the allylic/aromatic zone (6 to 8 ppm), the disappearance of the CA phenolic protons (9.13 and 9.52 ppm), and the appearance of a signal at 2.30 ppm for the –CH_3_ of the acetyl groups. The covalent linking of di-O-CA to Inu (di-O-CA-Inu) was confirmed by several changes: a slight shift in the allylic protons from 6.54 and 7.58 ppm in di-O-CA to 6.68 and 7.63 ppm in di-O-CA-Inu. Also, an overall broadening of the caffeate moiety signals (between 6.6 and 7.2 ppm) was observed due to the loss of mobility, while the signals of the 6 protons of the acetyl groups remained unchanged at 2.29 ppm. Moreover, the absence of the carboxylic proton was also observed, which was previously visible on CA and di-O-CA.

For the deprotected CA-Inu, the removal of the acetyl protection was confirmed by the disappearance of the 2.29 ppm signal, with the appearance of the phenolic protons at 9.13 and 9.58 ppm. Additionally, the signals for the allylic and aromatic protons reverted to a pattern similar to CA, with slight chemical displacement shift, peak broadening and absence of the carboxylic proton. Regarding the pattern of the Inu backbone, it remained stable throughout the synthesis, as can be observed for Inu, di-O-CA-Inu, and CA-Inu (Figure 2a). These experiments were carried out in d_6_-DMSO, which allowed the observation of signals from Inu (4.5 to 5.5 ppm), and CA and di-O-CA moiety (6–13 ppm), since d_6_-DMSO is an aprotic solvent.

#### 3.1.2. Attenuated Total Reflection Fourier Transform Infrared Spectroscopy (ATR-FTIR)

Infrared spectroscopy (Figure 2b,c) clearly indicated the presence of the caffeic moiety, despite the relatively low substitution degree. In Figure 2c, the *ν*ν C=O of the ester in CA-Inu appears at 1685 cm^−1^, while the *ν*ν C=C from the CA aromatic ring and allylic group appears at 1634, 1607 and 1517 cm^−1^. Full FTIR spectra for Inu, Mp(CA/Inu), CA-Inu, and CA is shown as Figure S2.

#### 3.1.3. Degree of Substitution

The DS of CA-Inu was determined using both ^1^H-NMR spectroscopy and the Folin–Ciocalteu assay. For the ^1^H-NMR spectra, the DS was calculated based on the ratio of aromatic to aliphatic signals from the caffeoyl moiety. The DS of CA-Inu was 0.07, as determined by ^1^H-NMR, and 0.08 using the Folin–Ciocalteu assay. The synthesis of similar compounds has been reported in the literature, such as totally substituted inulin cinnamate (DS 3.0) [28]; inulin *p*-hydroxycinnamate with different DS, ranging from 0.37 to 1.13 [30]; inulin vanillate with different DS ranging from 0.38 to 0.98 [30]; inulin ferulate with different DS ranging from 0.42 to 1.12 [29,30]. All these studies reported DS values are higher than that reported for CA-Inu in this work (DS 0.07).

### 3.2. Encapsulation of Caffeic Acid with Inulin

Microencapsulation of CA with Inu by spray-drying Mp(CA/Inu) was performed using a CCD with axial points. The influence of CA content (formulation variable) and inlet air temperature (operational variable) on the EE of CA was studied. Table 1 shows the experimental design for the encapsulation of CA with Inu by spray-drying, and the analysis of the variance (ANOVA) for EE of CA.

The EE of CA is an important parameter that represents the interaction of CA with Inu. The EE varied from 45.9% to 99.8% (Table 1), which were within the range of values reported in previous research for extracts containing CA. For example, spray-dried microparticles of spent coffee ground extracts had an EE of CA of 63% when inulin:maltodextrin (80:20 wt) was used as encapsulating agent [17]. A high EE of CA (86%) was reported for microparticles of green coffee extract with maltodextrin and skim milk from whole coffee fruit [43].

The ANOVA (Table 1) showed that both the linear and quadratic forms of CA and inlet air temperature, as well as the interaction between the CA and inlet air temperature, were significant (*p* ≤ 0.05) for the EE of CA. Moreover, 88.45% of the variability (R^2^ adj. for d.f.) was explained by the model with a non-significant lack-of-fit (*p* ≥ 0.05). Among the significant factors, the CA content had the greatest influence on the EE (*p* = 0.0005).

The response surface graph for the EE of CA with Inu (Figure S3) showed that EE was higher in microparticles with lower CA content. The Inu content was the same for all the experimental runs; therefore, the available Inu hydroxyl groups to form hydrogen bonds with CA increased as the CA content decreased, therefore increasing the EE. Additionally, the EE slightly increased when the inlet air temperature increased, which may be explained by the fact that higher inlet air temperature forms a crust more rapidly on the droplet surface, retaining CA and thereby increasing the EE. In addition, the X-ray diffraction pattern of the Mp(CA/Inu) with the lowest EE of CA showed diffraction peaks corresponding to crystalline CA (Figure S4). When the surface CA (non-encapsulated) was removed by washing with ethyl acetate:ethanol (2:1), these crystalline peaks disappeared, confirming that the EE decreased with lower drying temperatures and higher caffeic acid loads by CA crystallization in the feed solution. To maximize the EE, the optimal inlet air temperature was 167 °C, and the CA in 100 g of infeed was 0.39 g. Under these conditions, the model predicted 100% of EE.

### 3.3. Characterization of Microparticles of Caffeic Acid with Inulin and Caffeate-Inulin

Table 2 shows the physico-chemical and thermal characterization of CA-Inu and Mp(CA/Inu).

#### 3.3.1. X-Ray Diffraction

**Figure 3:**
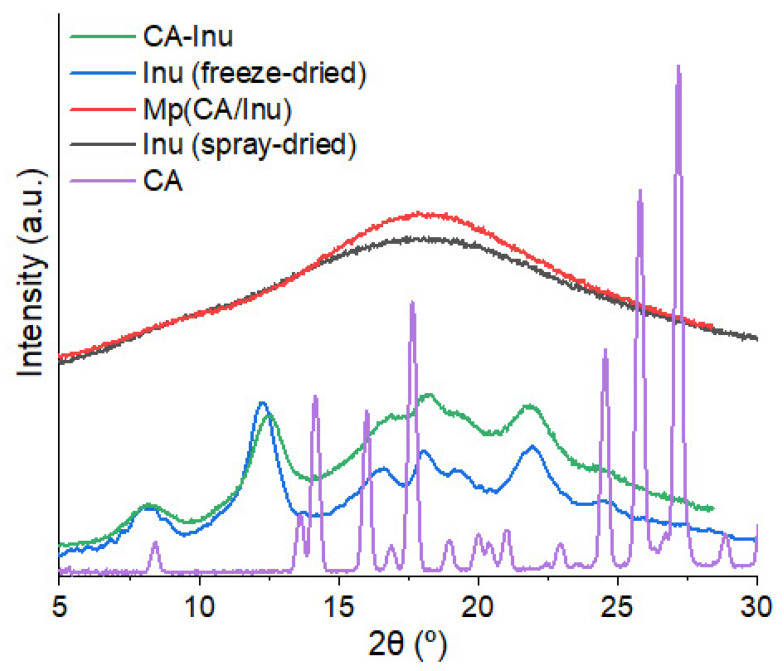
X-ray diffraction pattern for, CA, inulin caffeate (CA-Inu), Mp(CA/Inu), and spray-dried and freeze-dried inulin (Inu).

Figure 3 shows the diffraction pattern for CA, CA-Inu and Mp(CA/Inu). CA-Inu showed a semicrystalline pattern corresponding to Inu in its monohydrate crystal form [44]. On the contrary, Mp(CA/Inu) showed a typical amorphous diffraction pattern, without CA diffraction peaks, indicating that the CA was well dispersed in the amorphous Inu matrix. To assess the influence of CA on the diffraction pattern, spray-dried and freeze-dried Inu powders were prepared as controls. The crystalline structure of CA-Inu resembled that of freeze-dried Inu (8.2°, 12.2°, 16.5°, 18.1°, 19.2°, 22.0°, and 24.6°), and the characteristic peaks of CA were not observed due to the covalent bonding of CA to Inu. The almost identical crystalline structure indicated that the low DS (0.07) did not significantly affect the diffraction pattern. However, a higher amorphous content and broader peaks were noticed in CA-Inu, suggesting that the incorporation of CA in Inu negatively impacts crystal formation and growth. Mp(CA/Inu) and its control (spray-dried Inu) were amorphous, although the Mp(CA/Inu) showed higher amorphous content attributed to the dispersed CA.

#### 3.3.2. Thermal Behavior

**Figure 4:**
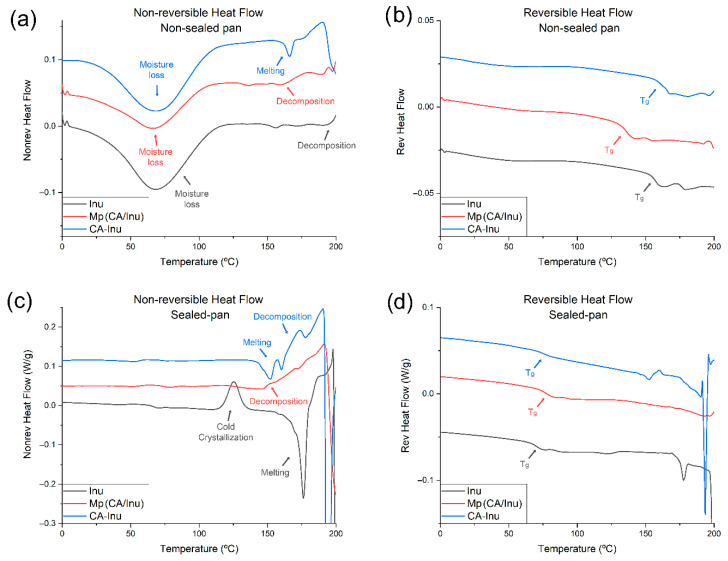
MDSC thermograms for caffeic acid/inulin microparticles (MP(CA/Inu)) and inulin caffeate (CA-Inu) and inulin (Inu). (**a**) Non-reversible heat flow non-sealed pan; (**b**) reversible heat flow non-sealed pan; (**c**) non-reversible heat flow sealed pan; (**d**) reversible heat flow sealed pan.

The thermal behavior of spray-dried Inu, Mp(CA/Inu), and CA-Inu was evaluated using the MDSC technique in non-sealed and hermetic aluminum pans (Figure 4a–d). The total heat flow of pure CA obtained by conventional DSC is provided in Figure S5, showing a melting peak at 217.97 °C, followed by an exothermic decomposition event. Since this transition lies outside the temperature range investigated for the Inu-containing samples, no further analysis was performed.

Non-hermetic condition: the samples measured in non-sealed pans should reflect the intrinsic behavior of the Inu without the plasticizing effect of moisture, since the water loss was observed as a broad peak ranging from 0 °C to 140 °C (Figure 4a) in the three samples. Spray-dried Inu showed a glass transition temperature (T_g_) of 157 °C (Figure 4b), which is consistent to the T_g_ values of 150 °C and 148 °C reported in the literature [45,46]. The incorporation of CA in the Inu matrix affected the T_g_ values, decreasing in the case of Mp(CA/Inu) to 136 °C, and increasing for CA-Inu to 162 °C. The decrease in T_g_ observed for Mp(CA/Inu) may be explained by the fact that the feed solution reached a pH ~3.5, which has been reported to hydrolyze Inu at the temperatures used [47]. However, the ^1^H-NMR of the spray-dried powder did not show monomeric fructose or a significant effect on the Inu signals. Despite this, a decrease in the molecular weight distribution of the chains cannot be ruled out, which could explain the observed decrease in the T_g_ temperature. For CA-Inu, the increase in T_g_ value can be attributed to the strengthened interactions between the phenolic moieties and the Inu hydroxyl groups through hydrogen bonds. Additionally, the dialysis process used for purification likely contributed to the T_g_ increase by removing low molecular weight chains that can permeate the membrane (MWCO 1000), thus shifting the molecular weight distribution to higher values. CA-Inu showed a melting event concurrent with the glass transition (Figure 4a,b), consistent with the crystalline peaks observed in its diffraction pattern (Figure 3). No cold crystallization was observed for CA-Inu and Mp(CA/Inu) (Figure 4b), which is expected since both Inu crystalline structures require crystallization water, which was lost by heating in open pans. While Inu monohydrate loses water to form the hemihydrate, the latter retains its crystallization water until its melting/decomposition. Spray-dried Inu and Mp(CA/Inu) did not show a melting peak and eventually showed a decomposition baseline drift, in agreement with the amorphous diffraction patterns.

Hermetic condition: In hermetic pans, no water loss was observed as expected (Figure 4c). The T_g_ was greatly reduced due to the moisture content of the powders and the well-known plasticizing effect of water on Inu [45,46] (Figure 4d). Spray-dried Inu showed an exothermic peak attributable to cold crystallization (Figure 4c), which was not observed for CA-Inu or Mp(CA/Inu), suggesting that this behavior is suppressed by the presence of CA. Regarding the melting process, Mp(CA/Inu) did not show a melting peak but instead exhibited a decomposition baseline drift, as observed under non-hermetic conditions. Spray-dried Inu showed an endothermic melting peak at 176 °C, arising from the melting of the cold crystallized fraction (Figure 4c). Finally, CA-Inu, despite not undergoing cold crystallization, showed a melting endothermic peak at 144 °C, confirming its semicrystalline nature as observed by XRD (Figure 3). While the melting of the cold crystallized spray-dried Inu (176 °C) was similar to the melting point reported for crystalline/semicrystalline Inu [45,46], the melting point of CA-Inu was considerably lower (144 °C). This behavior could be related to its isoforms previously reported [48], where Inu crystals are formed by helices of fructose residues. These showed higher melting points when the chains are longer, thus longer helices are formed. The CA substitution on the Inu chains could disrupt the helices packing, causing longer chains to behave as shorter chains with lower melting points.

#### 3.3.3. Morphology

**Figure 5:**
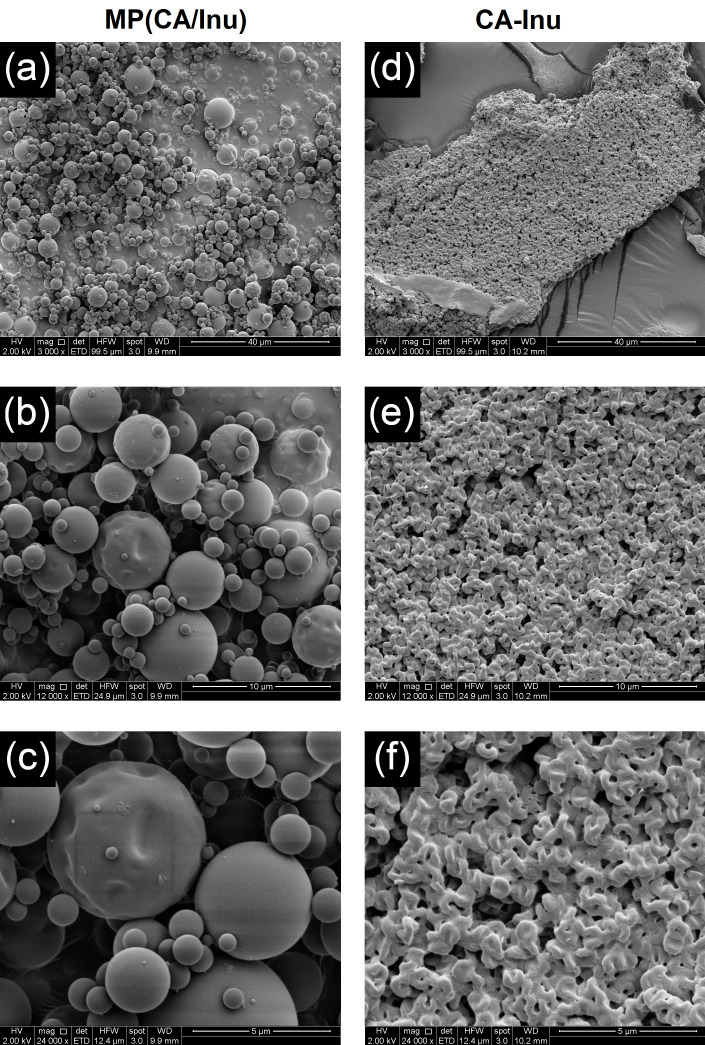
Scanning electron microscopic photographs for caffeic acid–inulin microparticles (Mp(CA/Inu)) and inulin caffeate (CA-Inu) captured at magnification (**a**,**d**) 3000×, (**b**,**e**) 12,000×, and (**c**,**f**) 24,000×.

Figure 5 shows the morphology and external structure of Mp(CA/Inu) (Figure 5a–c) and CA-Inu (Figure 5d–f). Mp(CA/Inu) displayed a spherical shape with some agglomeration, but not aggregate formation. Most microparticles had a smooth surface, and no cracks or holes were observed, consistent with the high EE of CA obtained. The particle size ranged from 1 to 10 µm, with an average particle size of 3.20 ± 0.17 µm, measured by laser diffraction. CA-Inu showed flakes (Figure 5d) with superficial spiral shapes and high roughness (Figure 5e,f), which can be attributed to the helical conformation of semicrystalline Inu [44]. The roughness may be attributed to channels formed during the sublimation of frozen water, which is influenced by the freezing conditions and the solid amount in the freeze-dried dispersion.

#### 3.3.4. Solubility, Moisture, Hygroscopicity, and Water Activity

The solubility in water was 88.1% for Mp(CA/Inu) and 100% for CA-Inu, respectively (Table 2). This difference in solubility may be explained by the physical state of the powders and the drying method used. Mp(CA/Inu) was in an amorphous state, which has been described to hydrate quickly [49]. However, spray-dried amorphous microparticles may form clumps when exposed to water, reducing their solubility [12,50]. In contrast, although CA-Inu had a semicrystalline state, it exhibited a more porous structure (Figure 5d–f) because this powder was lyophilized, resulting in a high surface area that facilitates and accelerates hydration upon contact with water [51].

The final moisture depends primarily on both the inlet and outlet air temperatures during spray-drying, as well as the amount and type of encapsulating agent [52]. Mp(CA/Inu) had a moisture content of 4.4% (Table 2), in agreement with other polyphenol/Inu spray-dried systems [53]. Mp(CA/Inu) was spray-dried at 167 °C, with an outlet temperature of approximately 92 °C, ensuring that the spray-drying conditions resulted in a powder with low moisture content. In contrast, CA-Inu had a moisture content of 7.6%, which is related to the drying method used (freeze-drying). In a comparison of different drying methods (spray-, freeze-, and vacuum-drying) for encapsulating sea buckthorn juice using Inu as the encapsulation agent [52], moisture contents of 2.62% and 4.75% were found for spray-dried and freeze-dried powders, respectively. This trend is similar to the results obtained for Mp(CA/Inu) and Inu-CAF in this study.

The hygroscopicity assay for Mp(CA/Inu) showed a 13.5% increase in weight due to water absorption after being exposed for 7 days at 81% relative humidity (saturated sodium sulfate), which is within the range observed for amorphous Inu spray-dried microparticles [12,45]. The absorbed water may be physically adsorbed on the particles or integrated into the crystalline structure. In contrast, CA-Inu exhibited a much lower hygroscopicity, with a value of 5.1%. Despite both powders being composed with Inu and CA, the formation of a new polymer with CA-functionalized Inu accounts for this difference.

Finally, the water activity (a_w_) was 0.189 and 0.253 for Mp(CA/Inu) and CA-Inu, respectively. These values were below 0.3, ensuring the inhibition of microbial growth [54].

#### 3.3.5. Antioxidant Activity

In ORAC measures, the antioxidant capacity is evaluated through a reaction between the antioxidants, a probe, and the oxygen-centered radicals generated by the thermal decomposition of AAPH. The oxygen-centered radicals react with a probe (fluorescein), leading to an oxidized product with different spectral behavior, allowing fluorescence detection. ORAC methodology has a priority hydrogen atom transfer mechanism, providing information on the stoichiometry of antioxidants [55].

Figure S6 shows the induction time (t_i_) for Trolox, CA-Inu, and CA at the same concentration (3.13 µM). As R_i_ depends on the AAPH concentration, and it was constant in the assay (18.75 mM), there is a direct proportional relationship between t_i_ and n values. The n value for Trolox has been previously reported as two [38]. Considering that the t_i_ of Trolox was 17.7 h, and that of CA-Inu and CA were approximately 1.6 and 2.4 times longer, respectively, CA-Inu and CA at 3.13 µM should be able to quench three and five molecules of the peroxyl radical. Although CA and CA-Inu contain the same number of –OH groups, the n value was lower for CA-Inu. In agreement with this result, the ORAC values (Table 2) of CA-Inu (21.0 ± 0.9 µmol Trolox/mg CA) were significantly (*p* < 0.05) lower than those of CA (28.0 ± 0.9 µmol Trolox/mg CA) and Mp(CA/Inu) (28.0 ± 0.5 µmol Trolox/mg CA), confirming that CA is completely available in the microparticles, whereas its antioxidant capacity decreased in the CA-Inu polymer. This reduction can be attributed to the covalent binding of CA to Inu in CA-Inu, which alters the resonance of the CA molecule, and, consequently, the availability of its hydroxyl groups, leading to a 25% decrease in its antioxidant capacity. Furthermore, the bulky moiety of Inu attached to the carboxylic group of the caffeic acid may produce a steric impediment, limiting the ability to quench peroxyl radicals [56].

### 3.4. In Vitro Simulated Digestion

**Figure 6:**
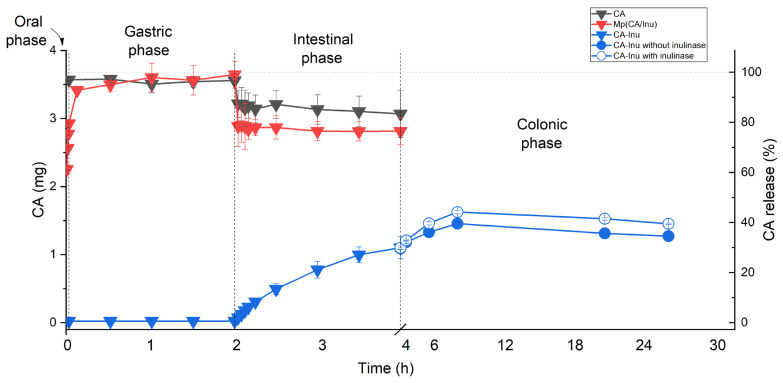
Caffeic acid in the digestion media (mg) and as a percentage of the initial CA content (%) for caffeic acid (CA), spray-dried caffeic acid–inulin microparticles (Mp(CA/Inu)), and inulin caffeate (CA-Inu). For the colonic phase, a sample without inulinase was assayed as a control.

The release profiles of CA from Mp(CA/Inu) and CA-Inu during in vitro simulated digestion are shown in Figure 6. CA was rapidly released from the amorphous Mp(CA/Inu), reaching 87% at the end of the oral phase. Although Inu can efficiently encapsulate phenolic compounds, it has poor retention in aqueous media, releasing water-soluble active compounds such as CA, even when Inu is neither dissolved nor metabolized [57]. CA levels remained practically constant during the gastric phase, since CA is stable under acidic conditions [58,59], and Inu is not degraded in the upper gastrointestinal tract. A slight decrease in CA was found at the beginning of the intestinal phase, which may be attributed to the binding of CA to the active site of pancreatic lipase by hydrogen bonds and π-stacking hydrophobic interactions [6]. At the end of the intestinal phase, around 80% of the CA from the Mp(CA/Inu) would be available for absorption. According to Olthof et al. (2001) [60], CA is absorbed up to 95% in the small intestine in humans, and therefore the colonic phase was not evaluated in the case of Mp(CA/Inu).

CA-Inu showed a different CA release profile compared to Mp(CA/Inu) (Figure 6). CA was not detectable after oral and gastric digestion of CA-Inu, indicating high stability of CA when covalently bonded to the Inu backbone, despite ester bonds being labile in acidic conditions. In contrast, CA-Inu underwent partial hydrolysis during intestinal digestion, resulting in a 30% release of CA, due to the enzymatic activity of the pancreatic lipase. In agreement with this, it has been reported that the ester bonds of esterified polyphenols (such as hydroxytyrosol acetate, hydroxytyrosol decanoate, hydroxytyrosol stearate) break when these compounds are subjected to the intestinal phase of digestion [61,62].

After the intestinal phase, the colonic phase was simulated both with and without inulinase (as a control) to assess the effect of Inu degradation on CA release from CA-Inu. A significant amount of CA still bound to Inu reached the colonic phase of digestion. The release of CA from CA-Inu increased up to 9 h of digestion, after which CA levels remained nearly constant until the end of the colonic phase. The release of CA from CA-Inu in the colonic phase was higher with inulinase than in its absence (Figure 6) as a consequence of the hydrolysis of the Inu backbone by the enzyme, as confirmed by thin-layer chromatography (Figure S7). Throughout the colonic phase, the appearance and gradual increase in fructose were observed, while the CA-Inu spot progressively faded until it nearly disappeared.

Therefore, based on these results, the chemical binding of phenolic compounds, as CA in this work, proved to be a successful strategy for protecting the bioactive compound during the oral and gastric phases of digestion. This approach enabled a partial and gradual CA release during the intestinal phase while still retaining a significant amount of the bioactive in the colonic phase, where further release was promoted by enzymatic degradation of the Inu backbone. This study highlights the potential of designing functionalized biopolymers with phenolic compounds to achieve targeted release at specific sites, such as intestine and colon, where prebiotic and polyphenol-related activities can take place.

## 4. Conclusions

Inulin caffeate (CA covalently bound to Inu, CA-Inu) and CA–inulin microparticles (Mp(CA-Inu)) were characterized and subjected to in vitro simulated gastrointestinal digestion. Whether CA was introduced into Inu by physical mixing or chemical conjugation, the thermal properties remained largely unchanged, except that cold crystallization was fully suppressed. However, it noticeably influenced the release profile of CA during the digestion phases. Although the Mp(CA-Inu) exhibited a high encapsulation efficiency of CA, it led to a rapid release of CA during the early stages of gastrointestinal digestion. In contrast, CA-Inu showed targeted delivery of CA, particularly during the intestinal and colonic phases. This finding suggests that covalent binding of phenolic compounds to biopolymers can modify their release profile, promoting targeted delivery in these later stages of gastrointestinal digestion. Chemical binding could be a valuable strategy that combines antioxidant and prebiotic properties, highlighting its potential as a functional ingredient for colonic targeting. Given the positive results obtained in this study, future experiments would explore other green synthesis routes more suitable for food applications to obtain CA-Inu. In addition, the functionalization of Inu with other bioactive compounds should be investigated, along with the evaluation of their release behavior. Furthermore, core–shell particle structures could be used to delay the rapid release of CA during the oral and gastric phases in the case of Mp(CA-Inu).

## Figures and Tables

**Table 1:** Composite central design for encapsulation of caffeic acid with inulin by spray-drying, and analysis of variance (ANOVA) for encapsulation efficiency.

Inlet Air Temperature (°C)	Caffeic Acid Content (g)	Encapsulation Efficiency (%)
127	0.27	97.1 ± 0.3
127	0.93	45.9 ± 01
193	0.27	99.1 ± 0.1
193	0.93	69.7 ± 0.4
160	0.20	98.2 ± 0.1
160	1.00	69.5 ± 0.4
120	0.60	86.3 ± 1.3
200	0.60	99.1 ± 0.6
160	0.60	98.0 ± 0.2
160	0.60	99.8 ± 0.1
160	0.60	93.5 ± 0.7
160	0.60	98.5 ± 0.1
Effect	Estimate	*p*-value
Constant	−32.92	
Caffeic acid content	2.12	0.0005 *
Inlet air temperature	1.64	0.0106 *
Caffeic acid content × Caffeic acid content	−110.53	0.0028 *
Caffeic acid content × Inlet air temperature	0.50	0.0284 *
Inlet air temperature × Inlet air temperature	−0.01	0.0202 *
Lack-of-fit		0.0595
R^2^ (%)	93.70	
R^2^ adjusted for degrees of freedom (%)	88.45	

* significant (*p* ≤ 0.05).

**Table 2:** Physicochemical and thermal properties of caffeic acid–inulin microparticles and inulin caffeate.

Parameters	CA-Inu	Mp(CA/Inu)	Inu
CA content (mg/g Inu)	64.5 ± 1.0 ^a^	35.2 ± 0.9 ^b^	-
EE (%)	-	98.4 ± 0.02	-
Solubility (%)	99.8 ± 0.2 ^a^	86.1 ± 0.6 ^b^	n.d.
Moisture (%)	7.6 ± 0.6 ^a^	4.4 ± 0.1 ^b^	7.3 ± 0.2
Hygroscopicity (g/100 g)	5.1 ± 1.1 ^a^	13.5 ± 0.3 ^b^	nd
Water activity	0.253 ± 0.001 ^a^	0.189 ± 0.004 ^b^	0.329 ± 0.002
Particle size (D_4.3_) (µm)	-	3.20 ± 0.17	5.11 ± 0.52
ORAC (TEAC, µmol Trolox/mg CA)	21.0 ± 0.9 ^b^	28.0 ± 0.5 ^a^	-
Thermal properties			
Sealed samples			
T_g_ (°C)	76.0 ± 1.1 ^b^	78.5 ± 1.1 ^a^	70.2 ± 0.9 ^c^
T_c_ (°C) onset (peak)	-	-	116.8 ± 1.2 (125.2 ± 1.5)
∆H_c_ (J/g)	-	-	−30.2 ± 1.3
T_m_ (°C) onset (peak)	144.6 ± 1.5 (152.3 ± 1.8)	-	176.2 ± 1.3 (176.2 ± 1.3)
∆H_m_ (J/g)	3.8 ± 1.8 ^b^	-	33.5 ± 3.3 ^a^
Unsealed samples			
T_g_ (°C)	166.2 ± 0.3 ^a^	136.8 ± 0.5 ^c^	157.1 ± 0.2 ^b^
T_c_ (°C) onset (peak)	-	-	-
∆H_c_ (J/g)	-	-	-
T_m_ (°C) onset (peak)	161.5 ± 0.2 (166.0 ± 0.3)	-	-
∆H_m_ (J/g)	4.8 ± 0.9	-	-

CA: caffeic acid; Inu: inulin; MP: microparticles, EE: encapsulation efficiency; T_g_: glass transition temperature; T_c_: crystallization temperature; ΔH_c_: crystallization enthalpy; T_m_: melting temperature; ΔH_m_: enthalpy for the melting transition; nd: not determined. Different letters indicate significant differences (*p* ≤ 0.05) between CA-Inu and Mp(CA/Inu).

## Data Availability

The original contributions presented in this study are included in the article and Supplementary Materials. Further inquiries can be directed to the corresponding author.

## Supplementary Materials

The following supporting information can be downloaded at: https://www.mdpi.com/article/10.3390/antiox15050591/s1. Figure S1: Full ^1^H NMR spectra of caffeic acid (CA), di-O-acetyl caffeic acid (di-O-CA), inulin (Inu), inulin di-O-acetylcaffeate (di-O-CA-Inu) and inulin–caffeate (CA-Inu). All spectra were obtained in d_6_-DMSO at 25 °C. Figure S2: Full FTIR spectra for inulin (Inu), CA–inulin microparticles Mp(CA/Inu), inulin–caffeate (CA-Inu), and caffeic acid (CA). Figure S3: Response surface graph for encapsulation efficiency of caffeic acid (CA) with inulin by spray-drying. Figure S4: X-ray diffraction patterns for caffeic acid–inulin microparticles (Mp(CA/Inu) with the lowest encapsulation efficiency obtained in the statistical design, before and after the removal of the non-encapsulated caffeic acid. Figure S5: Total heat flow of caffeic acid (CA) obtained by DSC. Figure S6: ORAC-FL profiles of (a) Trolox, (b) caffeic acid (CA), (c) inulin–caffeate (CA-Inu), and CA–inulin microparticles (Mp(CA/Inu)) at 3.13 µM. Figure S7: Thin-layer chromatography of the intestinal digestion after 2 h (I 2h), the colonic assay after 20 h without fructanase (C 20 h wo/F), the colonic assay after 20 h with inulinase (C 20 h w/F), and the colonic assay after 1 week with fructanase (C 1w w/F).

## Abbreviations

The following abbreviations are used in this manuscript:

CA	Caffeic acid
Inu	Inulin
CA-Inu	Inulin–caffeate
Mp(CA/Inu)	Spray-dried inulin microparticles
IBD	Inflammatory bowel disease
^1^H-NMR	Proton nuclear magnetic resonance
TTP	Ttriphenyl phosphine
DIAD	Diisopropyl azodicarboxylate
DMF	Anhydrous N,N-Dimethylformamide
di-O-CA-Inu	Inulin-3,4-diacetylcaffeate
di-O-CA	3,4-diacetylcaffeic acid
ATR	Attenuated Total Reflectance
DS	Degree of substitution
CCD	Central Composite Design
EE	Encapsulation efficiency
ANOVA	Analysis of variance
WAXS	Wide-angle X-ray scattering
MDSC	Modulated Differential Scanning Calorimetry
SEM	Scanning electron microscopy
ORAC	Oxygen radical absorbance capacity
AAPH	2,2′-Azobis(2-methylpropionamidine) dihydrochloride
*AOX*	Antioxidants
