# Supplementary material for: Caffeic Acid Release from Inulin Caffeate: A Comparative Study with Spray-Dried Inulin Microparticles Under Gastrointestinal Digestion
Source: Antioxidants (Basel). 2026 May 7;15(5):591. doi: 10.3390/antiox15050591 (PMC13203464; doi:10.3390/antiox15050591)
Supplement: Supplementary file 1 [file antioxidants-15-00591-s001.zip › antioxidants-4239322-supplementary.pdf]

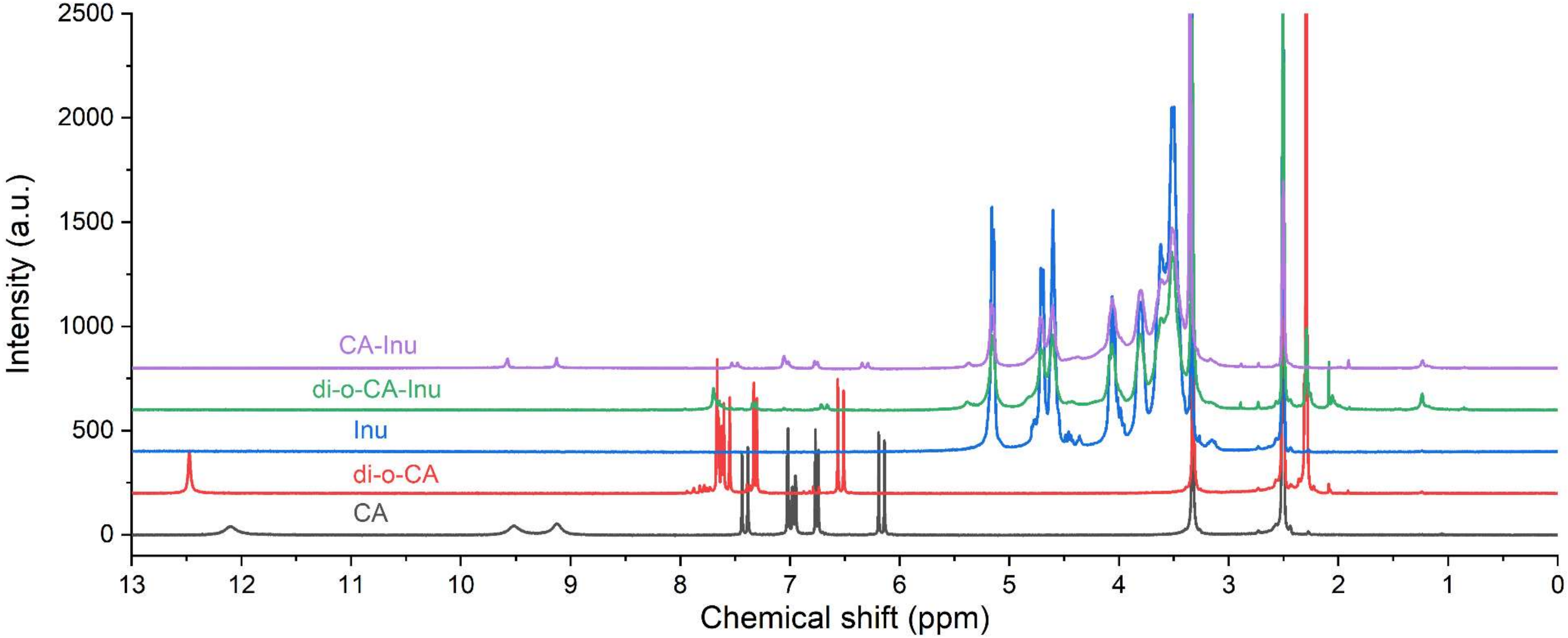

Figure S1. Full 1H NMR spectra of caffeic acid (CA), di-O-acetyl caffeic acid (di-O-CA), inulin (Inu), inulin di-O-acetylcaffeate (di-O-CA-Inu) and inulin caffeate (CA-Inu). All spectra were obtained in d6-DMSO at 25 ºC.

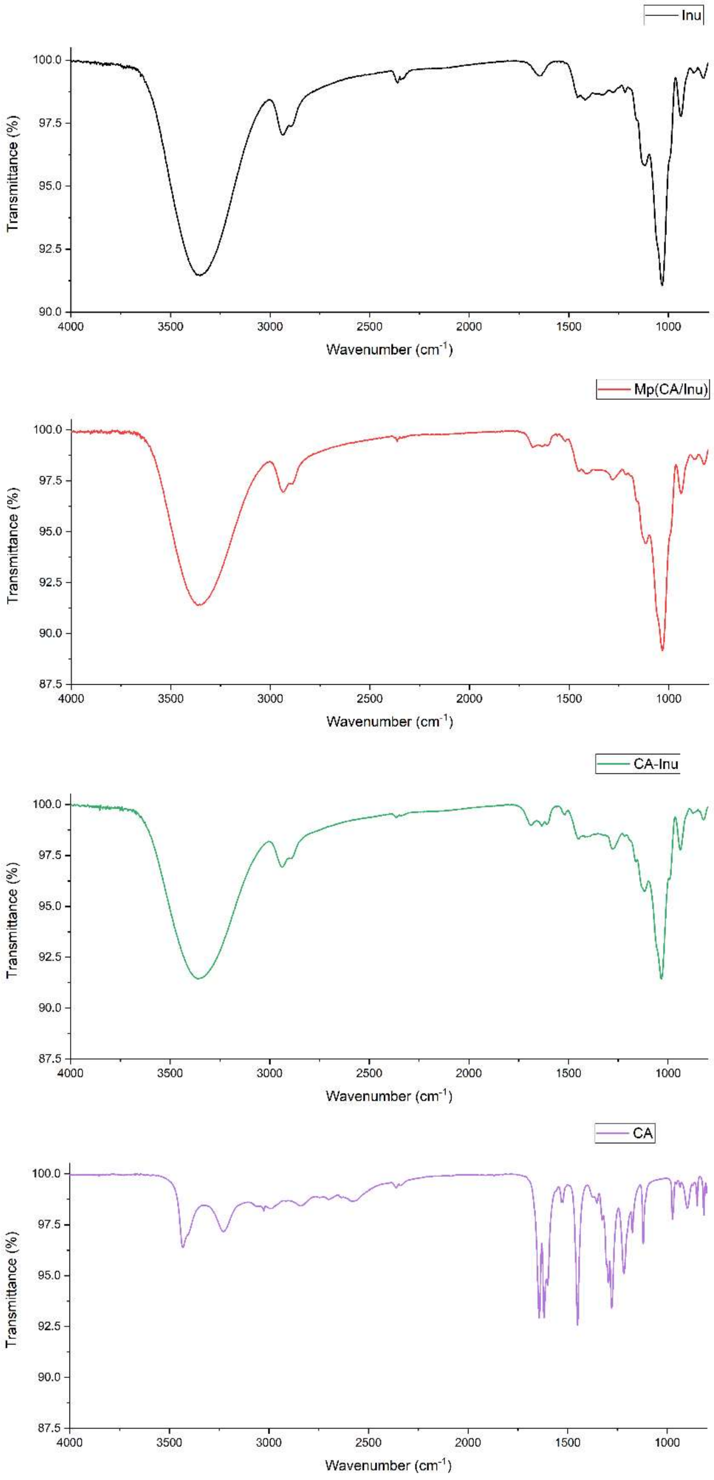

Figure S2: Full FTIR spectra for inulin (Inu), CA-inulin microparticles Mp(CA/Inu), inulin caffeate (CA-Inu), and caffeic acid (CA).

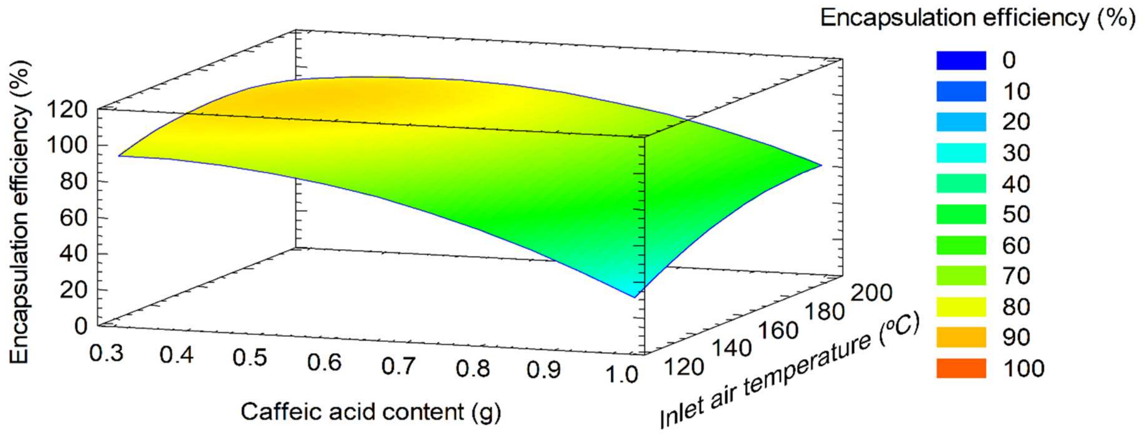

Figure S3. Response surface graph for encapsulation efficiency of caffeic acid (CA) with inulin by spray-drying.

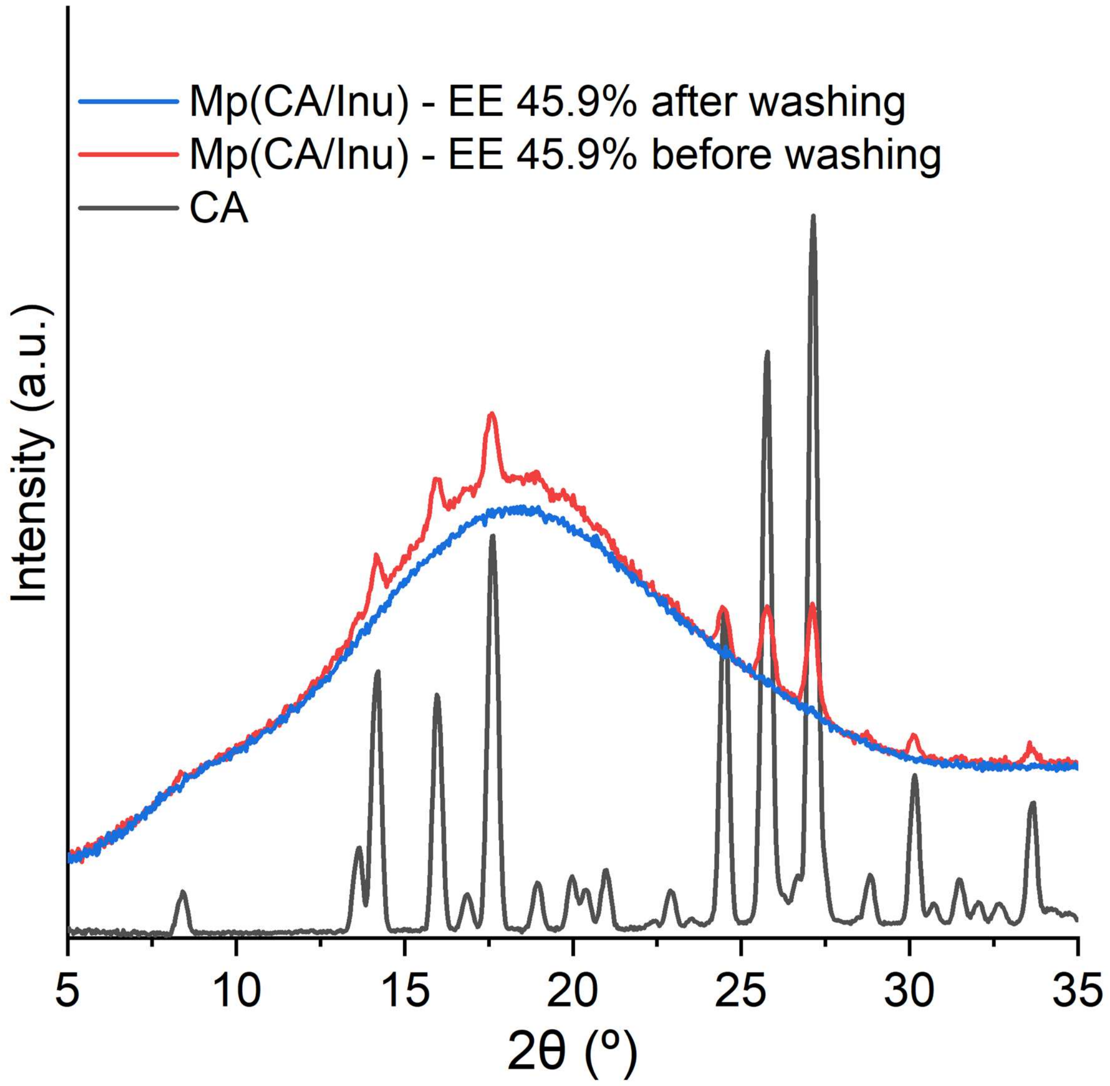

Figure S4. X-ray diffraction patterns for caffeic acid-inulin microparticles (Mp(CA/Inu) with the lowest encapsulation efficiency obtained in the statistical design, before and after the removal of the non-encapsulated caffeic acid.

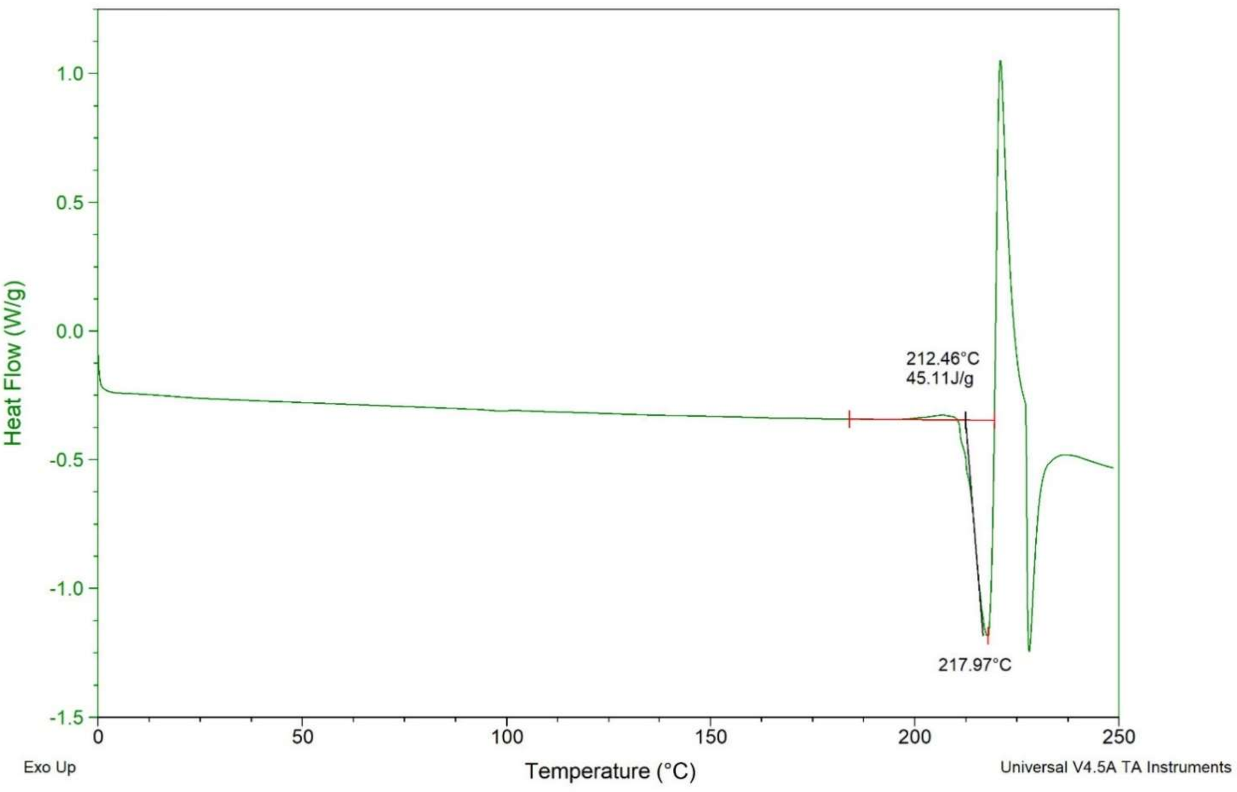

Figure S5. Total heat flow of caffeic acid (CA) obtained by DSC.

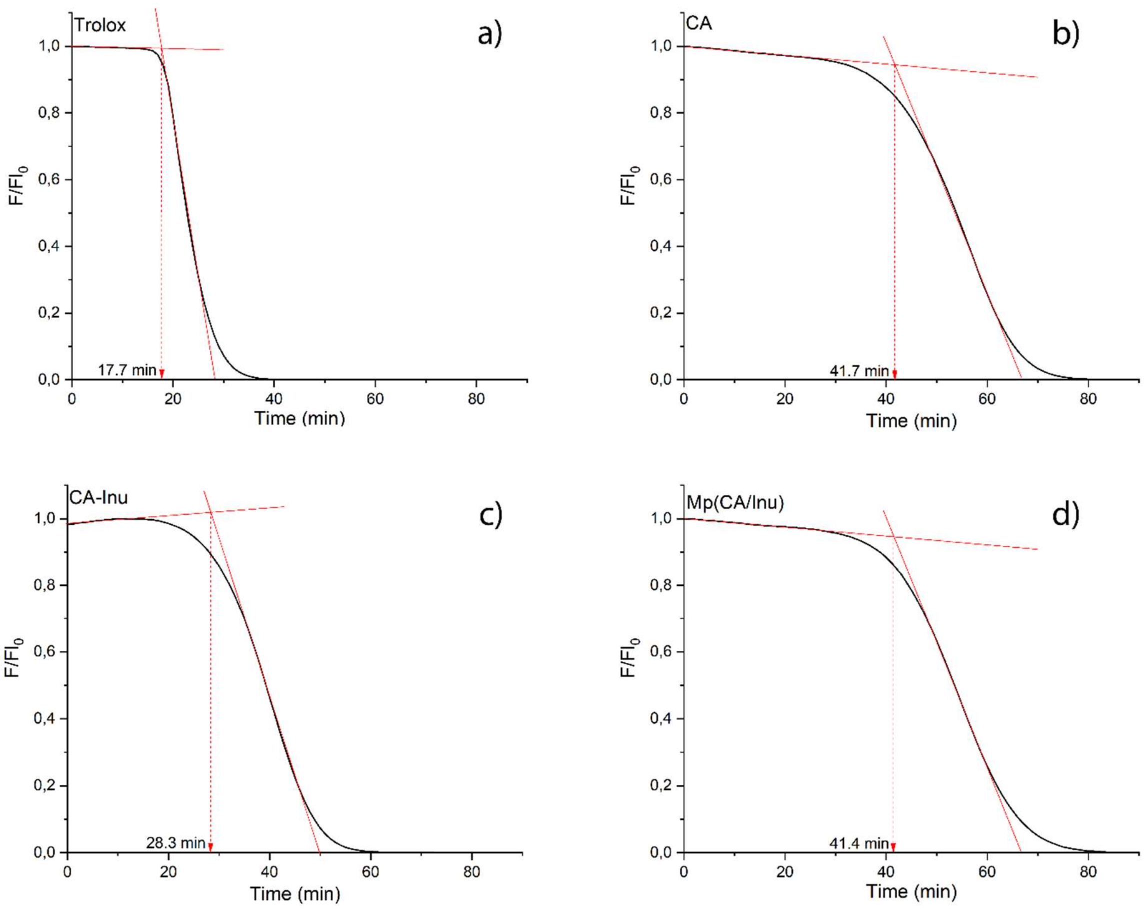

Figure S6. ORAC-FL profiles of (a) Trolox, (b) caffeic acid (CA), (c) inulin caffeate (CA-Inu), and CA-inulin microparticles (Mp(CA/Inu)) at 3.13 μM.

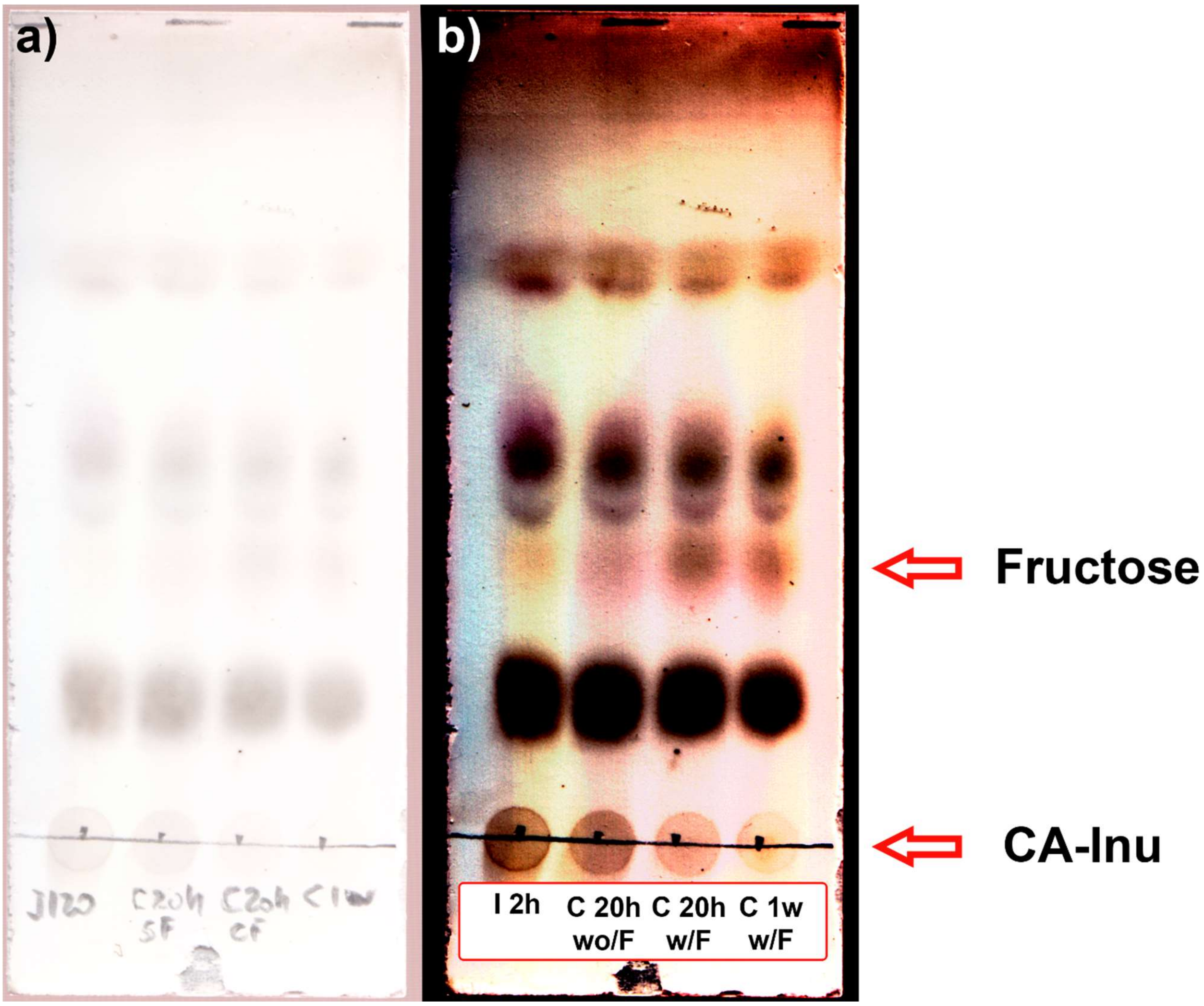

Figure S7. Thin layer chromatography of the intestinal digestion after 2 hours (I 2h), the colonic assay after 20 hours without fructanase (C 20h wo/F), the colonic assay after 20 hours with inulinase (C 20h w/F), and the colonic assay after 1 week with fructanase (C 1w w/F). The TLC was carried out in aluminum coated silica plates and developed in acetonitrile:water 85:15. The spots were stained with ethanol:sulfuric acid 10:90, pre-dried at 40 ºC for 5 minutes and then heated at 100 ºC for 5 minutes. a) Original raw scan (LIDE100, Canon) and b) photoshop enhanced image (gamma and curves). CA-Inu shows a RF of 0.00 since it does not elute, while fructose showed a RF of 0.34. The other spots come from the pancreatic lipase/bile salts present in the digestion media, while caffeic acid was not observed under these developing conditions at the concentration present (~0.03 mg/mL). A clear increase in fructose spot intensity and a decrease in CA intensity upon the use of inulinase provide evidence of enzymatic CA-Inu hydrolysis.
